# Sex disparities in STEMI pathophysiology: Presentation, treatments, and outcomes

**DOI:** 10.21542/gcsp.2026.1

**Published:** 2026-02-28

**Authors:** Akash Gopal, Swetha Reddy, George G. Kidess, Mohammad Akkawi, Timir K. Paul, Jawad Basit, M. Chadi Alraies

**Affiliations:** 1 Wayne State University School of Medicine, 540 E Canfield St, Detroit, Michigan, 48201, USA; 2 Ascension St. Thomas Hospital, 2000 Church St, University of Tennessee, Nashville, Tennessee, 37203, USA; 3 Rawalpindi Medical University, Tipu Road, 46000 Rawalpindi, Pakistan; 4 Detroit Medical Center, Cardiovascular Institute, 311 Mack Ave, Detroit, Michigan, 48201, USA

## Abstract

Despite the declining rates of ST-segment elevation myocardial infarction (STEMI) and improvements in care standards, significant disparities between sexes persist among patients diagnosed with STEMI. This review summarizes recent and relevant literature that highlights the differences between men and women concerning STEMI pathophysiology, risk factors, clinical presentation, treatment protocols, diagnostic challenges, and outcomes. Many of these disparities interact, resulting in poorer outcomes for female patients with STEMI. However, there is hope through the adoption of evidence-based treatment plans.

## Introduction

Despite a steady decline in the global incidence of ST-segment elevation myocardial infarction (STEMI) over the past few decades, this condition continues to be a significant contributor to cardiovascular mortality worldwide. In unadjusted analyses, women diagnosed with STEMI experience worse outcomes than men, with nearly double the in-hospital mortality (8% vs 4%); however, this difference is attenuated after adjustment for age and baseline clinical characteristics^1^. These findings are supported by a systematic review of the literature, which similarly demonstrated that observed sex-based differences in STEMI outcomes are largely explained by differences in age and comorbidity burden at presentation^2^. However, there are a multitude of factors contributing to persisting disparities in outcomes observed in female populations with STEMI. This selective narrative review, based on the authors’ expertise, aims to identify the differences in mortality rates between men and women diagnosed with STEMI, and to explore the underlying reasons for these disparities by reviewing relevant literature.

## Pathophysiology

**Figure 1.**
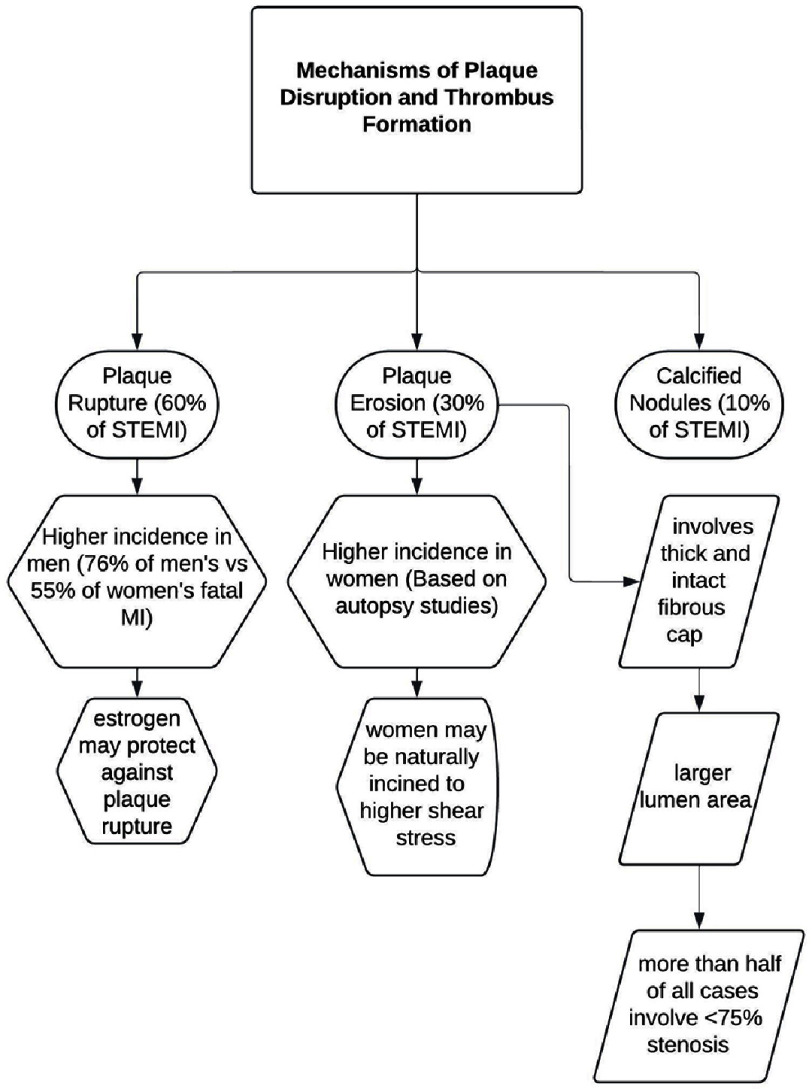
Mechanisms of plaque disruption and thrombus formation.

Myocardial infarction (MI) is a clinical condition characterized by a sustained loss of blood flow, leading to ischemia and the death of cardiac muscle cells^3^. There are two main types of MI based on electrocardiogram (EKG) results: ST-segment elevation myocardial infarction (STEMI) and non-ST-segment elevation myocardial infarction (NSTEMI)^4^. This literature review focuses on STEMI, which occurs when a thrombus completely occludes an artery supplying the infarcted area. The location of the ST-segment elevation on the EKG indicates the area of the heart that is affected^4,5^. The most common mechanisms leading to plaque disruption and subsequent thrombus formation include plaque rupture, plaque erosion, and calcified nodules (Figure 1)^6^.

Differences in the pathophysiology of STEMI between men and women become evident when analyzing specific age groups. For instance, premenopausal women tend to have a higher risk of plaque erosion compared to postmenopausal women^6,7^. Women of all ages experience STEMI from plaque erosion at higher rates than men, whose primary STEMI concern is plaque rupture^7^. Plaque erosion is a distinct mechanism of STEMI, characterized by a thick and intact fibrous cap and larger lumen area. While optical coherence tomography (OCT) studies show that plaque erosion accounts for ∼25% of STEMI cases, more than half of all cases of plaque erosion present with less than 75% stenosis^8^. This finding demonstrates that erosions can occur regardless of luminal stenosis^8^.

Several theories explain these biological differences. First, research has shown that estrogen possesses anti-inflammatory and vasoprotective properties, as well as the ability to promote endothelium-independent dilation of coronary arteries^9^. These effects help stabilize plaques in blood vessels, thereby reducing the likelihood of rupture. Second, plaque erosion is influenced by shear stress within blood vessels, with higher shear stress correlating to increased plaque erosion^10^.

Anatomical differences between male and female hearts may also play a role. Women naturally exhibit several biological traits that may predispose them to greater shear stress, including smaller coronary artery diameters and increased cardiac blood perfusion^11^. The reduced diameter of women’s coronary arteries often results in more frequent angina^12^.

Furthermore, data from large observational registry studies indicate that women are more likely than men to present with acute coronary syndrome (ACS) due to non-obstructive coronary artery disease, with prevalence rates of 10.5% for women compared to 3.4% for men^13^. Recognizing the different profiles of these diagnoses is essential for optimizing patient care. Conditions such as coronary vasospasm and spontaneous coronary artery dissection can present similarly to STEMI, often featuring angina and ST-segment elevations on EKG.

## Epidemiology and demographics

Cardiovascular disease continues to be one of the leading causes of death worldwide. In the United States, statistics show that a heart attack (myocardial infarction, or MI) results in a death approximately every sixty seconds, despite a decline in incidence rates over time^14,15^. However, the risks associated with MI are not uniform across all demographics; they vary significantly based on age and gender. A recent systematic review reported that the global prevalence of MI is 3.8% in individuals under 60 and 9.5% in those over 60^16^. Furthermore, when gender is factored in, MI tends to affect more men than women, with prevalence rates sometimes being 3–5 fold higher, indicating a clear disparity^15,16^. While the exact rationale for this difference is not entirely understood, some researchers hypothesize that certain risk factors, such as smoking, are more prevalent among men, which may contribute to their higher rates of MI^17,18^. Additionally, previous studies have indicated that estrogen has beneficial impacts on cardiac perfusion^9^. However, while hormonal factors may influence cardiovascular risk, they are unlikely to account for the broader sex-based disparities observed in STEMI incidence, management, and outcomes. Consistent with this, prior studies have reported that the incidence of STEMI is more than double in men compared to women^17,18^.

The number of patients presenting with STEMI has decreased significantly over the last thirty years. This decline is partly due to comprehensive public health policies and increased awareness of STEMI risk factors^18,19^. STEMI results from arterial blockage caused by atheroembolic processes, meaning that the risk factors for generalized coronary artery disease are also risk factors for STEMI^18^. These include smoking, hypertension, increased body mass index (BMI), diabetes mellitus, dyslipidemia, congestive heart failure, and valvular disease^20–22^. Patients with one or more of these risk factors are much more likely to experience an MI, particularly a STEMI.

Recent research has also linked sustained elevated levels of inflammatory markers and specific genetic polymorphisms to the development of STEMI^21,22^. While the patient population suffering from STEMI is very diverse, risk factors such as those previously mentioned help to paint a more accurate patient profile. Younger patients with STEMI ( ≤ age 50), for example, are more typically Caucasian males, with high rates of tobacco use and a family history of coronary artery disease^23^. An excessive reliance on demographic patterns, combined with different patient presentations between men and women, may delay recognition or contribute to misdiagnosis^24^.

### Clinical presentation and symptoms

Before discussing disparities between men and women with STEMI, it is essential to examine differences in symptom presentation between the two groups. These differences in presentation can lead to variations in diagnosis rates and the aggressiveness of interventions^25–27^. Typically, MI is characterized by an intense, crushing chest pain, which forms the basis for early recognition and diagnosis^28,29^. A systematic review and meta-analysis of observational studies demonstrated that women are less likely to present with classic chest pain compared with men (88.3% vs 92.4% among younger patients), consistent with prior studies^25–27,30^. Instead, women experiencing STEMI or MI more broadly tend to exhibit what clinicians refer to as “atypical” symptoms. These can include shortness of breath, pain between the shoulder blades, back pain, nausea and vomiting, and heart palpitations^26,27,30^.

The 2021 ACC/AHA chest pain guidelines have removed the term “atypical chest pain” in an effort to diagnose coronary artery disease or MI earlier and more inclusively^31^. Furthermore, recent data also support the finding that women with STEMI experience more clearly defined prodromal symptoms compared to men, such as fatigue^32^. Notably, many of these symptom differences decrease as both sexes age, although the reasons for this change remain poorly understood^33^.

Furthermore, registry-based data indicate that women generally present with multiple comorbidities when experiencing STEMI, including heart failure, renal failure, diabetes mellitus, hypertension, and dyslipidemia^34^. While some of these conditions affect both sexes, diabetes, for instance, increases the risk of coronary artery disease and MI in women by a factor of two compared to men^35,36^. The adverse effects of these conditions on women are compounded, leading to poorer outcomes in STEMI, which will be examined later in this review^37^.

Symptom presentation is inherently subjective and may differ based on patient reporting and provider documentation. Additionally, various studies have reported that these observed sex differences are attenuated after adjusting for age and existing comorbidities. “Atypical” symptoms are uniquely defined across studies and can also be heterogeneous.

## Treatment disparities

### Gender bias and misattribution of symptoms with psychological causes

Historically, women’s physical health complaints have often been misattributed to psychological causes, reflecting gender bias in medical diagnosis and treatment^12,38^. This pattern has contributed to the delayed recognition of serious medical conditions in women and highlights the need for continued re-evaluation of how women’s health concerns are perceived in a clinical setting.

Existing literature suggests women are more likely to report symptoms of anxiety and related somatic complaints, including cardiac, pulmonary, and gastrointestinal dysfunction, such as dyspnea and nausea^38^. This overlap in symptom presentation may contribute to diagnostic uncertainty, increasing the risk that women’s cardiac symptoms are misattributed to psychological causes rather than recognized as manifestations of acute coronary syndromes.

Gender bias is difficult to measure objectively and is often inferred from observed patterns in delivery and patient outcomes. Bias alone may not be the sole cause of disparities; various structural, system-wide, and biological factors may also play a role. Thus, further research is necessary in order to differentiate these mechanisms.

### Percutaneous Coronary Intervention

Currently, the gold standard treatment for STEMI is primary percutaneous coronary intervention (PCI). This is an invasive, yet non-surgical procedure aimed at reducing arterial blockage and restoring perfusion to cardiac tissues deprived of oxygen^39–41^. The effectiveness of PCI is well established, particularly when it is provided within an adequate time frame. The widespread standardization of this intervention in STEMI care plans has significantly improved patient prognosis over the past decade^41,42^.

PCI is so integral to STEMI treatment that a patient’s door-to-balloon time (D2B) has become a key measure of their treatment quality; shorter D2B times are associated with better patient outcomes. However, if PCI cannot be administered promptly, specifically if there is a delay of more than two hours from the first medical contact to potential PCI, or if patients are taken to a hospital without PCI capabilities, fibrinolytic therapy is recommended^41,43^. These reperfusion methods, which have standardized administration guidelines in both Europe and America, are crucial for adequately treating patients experiencing STEMI^43,44^.

### Gender discrepancies in delivery of STEMI treatment

Despite the proven effectiveness of rapid PCI for populations diagnosed with STEMI, research shows that the implementation of this therapy varies between men and women^45^. For instance, the time until first medical contact is generally longer for women compared to men, with reported delays averaging more than 40 min, which reduces the likelihood of timely reperfusion and receipt of PCI.

Additionally, when PCI is performed, women often experience longer door-to-balloon (D2B) times, further diminishing the effectiveness of reperfusion therapy^6,46^. While early studies have suggested that women delay seeking care due to increased hesitation or misattribution of symptoms, this viewpoint has largely been challenged. Recent evidence has shown that prehospital delays among women are more likely to be attributed to a higher prevalence of “atypical” symptom presentation, reduced recognition that symptoms represent an acute myocardial infarction, and lower perceived personal cardiovascular risk, rather than patient-level hesitation alone^47^.

Across all age groups, women are generally less likely to receive certain treatments such as PCI, coronary bypass surgery, and even fibrinolytics, a disparity that persists even after adjustment for age (e.g., adjusted odds ratio for PCI 0.74, 95% CI [0.72–0.76])^45^. Despite some promising quality improvement measures, significant sex differences in reperfusion therapies continue to exist^48^. In addition to PCI, women are statistically less likely to receive therapies such as aspirin, clopidogrel, unfractionated heparin, enoxaparin, and beta blockers; for example, aspirin was administered in 96.0% of women compared with 97.8% of men, with similar gaps observed for other guideline-directed therapies (*p* < .001)^49^. This trend is consistent for both older and younger women^46^.

Furthermore, although men and women tend to adhere to treatment protocols at an identical rate, women under the age of 55 remain significantly undertreated in the period after an MI, even after accounting for age and comorbid conditions. Smolina et al. attributed this disparity to lower rates of treatment initiation among women in younger age groups, rather than differences in adherence^50^. Clinical guidelines currently recommend beta-blockers, statins, and ACE inhibitors for all patients after experiencing an MI. However, younger women were significantly less likely to be prescribed adequate therapy within 2 months of discharge compared with age-matched men (adjusted OR 0.72, 95% CI 0.57–0.91)^50^.

This finding indicates important implications for secondary prevention and long-term cardiovascular outcomes. At the end of 1 year, roughly 33% of all acute MI patients received optimal therapy. The largest gap in care remained among women in younger age groups. This finding further emphasizes the importance of post-discharge care as a pivotal window that can shape long-term outcomes for MI patients. Thus, the need for standardized post-discharge protocols is critical, and attention should be paid to the increased cardiovascular risks in the young female demographic^50^.

Many studies describing treatment disparities rely on large registry-based data, which may remain subject to residual confounding despite multivariable adjustment. In addition, the magnitude of observed disparities may vary by health system and over time.

### Contributing factors

The reasoning for these disparities is not entirely clear. These differences may reflect variation in STEMI presentation in women and differences in physician response. “Atypical” symptoms, older age, and more serious comorbidities, such as diabetes, might make women appear to be less suitable candidates for invasive therapies^51^. While innate biology and differing mechanisms of STEMI pathophysiology between men and women may play a role in the condition’s development and diagnosis, other factors, such as physician bias, are postulated to play a significant role in treatment disparities as is evidenced by the fact that evidence-based treatment disparities persist even after adjustment for age and comorbid conditions^45,48,52^.

## Outcomes and complications

There is a broad spectrum of complications that can occur after MI, including papillary muscle rupture, ventricular septal rupture, arrhythmias, aneurysms, and ventricular remodeling that can ultimately result in heart failure. Each of these complications can have both short-term and long-term outcomes. Various pericardial syndromes can occur, including peri-infarction pericarditis and Dressler syndrome, an autoimmune reaction where antibodies cause an inflammatory reaction, leading to friction rub, pleuritic chest pain, and other complications. Managing post-MI complications and outcomes is critical and can improve patients’ quality of life^53^.

When considering all the previously discussed metrics, it is not surprising that women with STEMI experience higher in-hospital and post-hospitalization mortality rates than men in unadjusted analyses (e.g., in-hospital mortality 11.1% vs 6.8%; unadjusted OR 1.72, 95% CI 1.67–1.77)^45,54–56^. Findings such as this stress the clinical impact of cumulative delays and treatment disparities across the care continuum. Interestingly, mortality rates are even higher in women of minority backgrounds, such as Hispanic women^54^. Women receive revascularization less often than men, and this disparity is more pronounced among younger women; for example, in large national cohorts, women were significantly less likely to undergo revascularization procedures such as PCI or CABG compared with men (e.g., PCI performed in 62.9% of Caucasian women vs 67.0% of Caucasian men, *p* < 0.001), a difference that persists after adjustment for age and comorbidities^54^. These findings may contribute to differences in myocardial salvage and short-term survival.

Women also experience several complications during STEMI care, including postoperative bleeding, the need for blood transfusions, acute ischemic stroke, and vascular complications; for example, women experienced higher rates of postoperative bleeding compared with men (6.6% vs 5.2%; unadjusted OR 1.29, 95% CI [1.24–1.34])^6,45^, which has important implications for periprocedural safety and recovery. Long-term mortality rates for women with STEMI are worse, particularly at one month and three years post-event, especially when complicated with cardiogenic shock^35,57^. However, long-term follow-up data from a post hoc subanalysis of a randomized controlled trial (EXAMINATION-EXTEND) suggest that sex-based mortality differences attenuate over time, with disparities diminishing by roughly 10 years after the index STEMI event^58^.

A substantial proportion of observed outcome differences likely reflects older age at presentation among women with STEMI. After age adjustment, many mortality disparities between men and women are markedly reduced; however, age adjustment does not fully eliminate differences among younger women, in whom higher mortality and complication rates persist^35^.

Unfavorable events in this population are largely the result of pathophysiological differences in STEMI etiology, differences in symptom presentation between genders, and delays in healthcare response following symptom onset. These factors contribute to women being diagnosed with STEMI less frequently than men, leading to a lower likelihood of receiving first-line therapies^6,7,45,59^. Therapies like PCI offer the best prognosis for those with STEMI when administered promptly^41,42^.

In large prospective cohort studies, delays in access to healthcare and delivery further contribute to increased risks of adverse clinical events for women during the first year following the onset of symptoms. In a large prospective cohort study, the VIRGO study highlighted that women had significantly longer pre-hospital delays than men. From symptom onset to hospital arrival, the time was 54 min longer for women^59^. Among patients who received reperfusion therapy, only 58.9% of women received it in a timely manner. For primary PCI, 41% of women missed the 90-minute D2B target when compared to 29% of men (*p* < 0.001); consistent with findings from registry-based cohort analyses, female sex is independently associated with longer D2B times (ratio of geometric means 1.13, 95% CI 1.02–1.26)^59,60^. Delays of this magnitude are clinically significant given the strong association between prolonged ischemic time and increased infarct size and mortality. While the differences between men and women with STEMI can vary, the overall effect on outcome remains clear.

While individual cohort studies and registry analyses report higher crude mortality and complication rates among women with STEMI, these differences are often attenuated after adjustment for age and baseline clinical characteristics. Several analyses have demonstrated no independent association between sex and mortality after accounting for these confounding factors.

### The role of age in sex disparities

Age is of significant importance when discussing the sex disparities manifested in those who have suffered STEMI. For example, while it is true that women as a group have higher crude (unadjusted) mortality rates following STEMI, these inequalities gradually dissipate following stepwise adjustment for age^45^. Indeed, the only population in which they appear to persist is younger women aged 19–59, even after age adjustment^45^. Interestingly, according to recent data from the National Inpatient Sample, these same trends persist when examining certain post-STEMI outcomes such as vascular complications and major bleeding events, with disparities between men and women reducing as the cohort increases in age^61^. Conversely, however, disparities in treatment, namely women being less likely to receive fibrinolytic therapy or PCI, is something that persists even after accounting for age^45^.

Sex-based disparities in younger patients remain largely understudied. Subgroup definitions and age cutoffs vary across studies, and as a result, disparities between different age groups should be interpreted with caution.

### Implications for young women with STEMI

Younger women (<55 years) represent a vulnerable subgroup within the STEMI population. While hospitalizations attributed to STEMI have declined over time, the number of women who are aged <45 years are increasing, and in-hospital mortality rates have remained stagnant for more than a decade^62^. Women in younger age groups (18–34) continue to experience disproportionately higher rates of in-hospital mortality, cardiogenic shock, and mechanical complications as opposed to women from older age groups. This is despite a lower burden of typical cardiovascular risk factors^62^.

In addition, younger women face unique gaps in treatment that are potentially contributing to adverse outcomes. Previous literature has attributed this to delayed recognition of symptoms, underestimation of cardiovascular risk, and a lower likelihood of receiving guideline-directed therapies in women presenting with an acute MI^63^. Younger women are more likely to face non-atherosclerotic mechanisms of MI, including spontaneous dissection of the coronary artery, coronary vasospasm, and microvascular dysfunction, which may increase diagnostic uncertainty and hesitancy in acute care settings^63^. Thus, these findings highlight the importance of vigilance in clinical settings and of advocating for strict adherence to STEMI protocols to address persistent care gaps in younger women.

## Future directions and research gaps

Future work should examine outcomes in uniquely vulnerable populations. In persons suffering from comorbid mental health disorders, for example, recent evidence demonstrates that depression, anxiety, and post-traumatic stress disorders are increasingly prevalent after an MI. Specifically, these disorders are more prevalent amongst women and young patients. However, during hospitalizations for cardiovascular reasons, these disorders are rarely screened^66^. This topic introduces a discussion of whether or not psychological history plays a role in sex disparities in STEMI outcomes. Further research in female populations is necessary to address this gap, which predominantly focuses on males.

In addition, recent advancements in artificial intelligence-informed triage and risk-based decision support systems have potential to decrease delays in cardiovascular procedures for patients who present to the hospital with chest pain. The topic of digital health tools in the diagnosis and treatment of STEMI is highly relevant in how this may affect women^67^. Furthermore, continuing to follow STEMI outcomes over longer timelines may provide clarity in how sex-based disparities evolve over time, and can inform the development of more standardized care protocols.

## Limitations

As a selective narrative review, this paper’s conclusions are subject to certain limitations. The included literature, for example, was drawn from the author’s expertise rather than a more systematic search strategy with inclusion and exclusion criteria, which introduces a lack of reproducibility. Furthermore, although the studies discussed were heterogeneous and drawn from high-quality, contemporary sources, it is possible that some relevant results were excluded.

## Conclusion

In patients with STEMI, there are significant sex differences in the condition’s pathophysiology, clinical presentation, demographic characteristics, and treatment delays, all of which contribute to poorer outcomes for women. These disparities (Table 1) have persisted over time in unadjusted analyses, with age accounting for a substantial proportion of observed differences; however, important gaps remain, particularly among younger women.

**Table 1:** Sex disparities in epidemiology and demographics, clinical presentation and symptoms, treatment disparities, and outcomes and complications of STEMI.

Category	Men	Women
Epidemiology and Demographics	• Higher prevalence of MI and STEMI • Often attributed to lifestyle factors such as smoking • More likely to present at a younger age	• Lower prevalence of MI and STEMI compared with men • Protective effects of endogenous estrogen • More likely to present at an older age
Clinical Presentation and Symptoms	• Classic symptoms: intense, crushing chest pain	• “Atypical” symptoms: shortness of breath, back pain, nausea, fatigue • Symptoms may lead to later diagnosis • Higher prevalence of prodromal symptoms
Treatment Disparities	• More likely to receive primary PCI • Shorter door-to-balloon times • Higher likelihood of receiving evidence-based medications (aspirin, clopidogrel, etc.)	• Less likely to receive primary PCI and other invasive procedures • Longer door-to-balloon times • Often under-treated with evidence-based medications • Potential hesitation in seeking care, leading to treatment delays
Outcomes and Complications	• Lower in-hospital mortality rates and better long-term survival • Fewer complications overall	• Higher in-hospital and post-discharge mortality • Increased risk of bleeding, vascular complications and stroke

However, there is hope for change through the standardized adoption of more evidence-based approaches that ensure equal and timely access for women to treatments such as PCI. A recent study conducted at the Minneapolis Heart Institute, for example, demonstrated that implementing a standardized, PCI-based STEMI protocol successfully reduced treatment disparities between men and women^64^. In this study, women were older and had a greater number of comorbidities, including hypertension, diabetes, and cardiogenic shock, but had a lower likelihood of having associated coronary artery disease or smoking history. After age adjustment, the difference in mortality between genders was no longer significant, and a five-year follow-up showed no significant difference in survival between sexes^64^.

This work was further supported by a study by Huded et al. in the Journal of the American College of Cardiology, which proposes a 4-step protocol that has already demonstrated promising results^65^. The protocol itself involves implementing the following changes: (1) emergency department rapid catheterization lab activation, (2) a STEMI safe handoff checklist, (3) immediate patient transfer to an immediately available catheterization lab, and (4) a radial first approach to primary PCI^65^. Results from a prospective, observational, registry-based study demonstrated that implementing this 4-step protocol successfully reduced disparities in guideline-directed medical therapy, D2B times, and in-hospital adverse events^65^.

A variety of barriers, however, make changes such as these difficult to implement. On a patient level, for example, alternative presenting symptoms that women experience at the onset of STEMI can make recognizing the condition itself as a cardiovascular emergency more difficult, further contributing to healthcare delays^47^. This barrier extends further when considering the potential for provider misattribution of symptoms, which also leads to delays in guideline-directed medical therapy^25–27,47^.

Additionally, there are standard logistical difficulties in translating results from a single-center protocol study across a variety of healthcare environments worldwide. It is well known that change in healthcare settings takes time, especially in larger and more diverse settings. A robust communication and organizational framework is necessary to carry out such a task, and many centers may not have the required resources. Widespread adoption of these standardized protocols, however, may effectively reduce sex disparities in STEMI and continue to improve patient outcomes both in the short and long term.
